# Comparative analysis of chloroplast genome of *Lonicera japonica* cv. Damaohua

**DOI:** 10.1515/biol-2022-0984

**Published:** 2024-11-11

**Authors:** Jiaqiang Zhang, Huichun Liu, Wenting Xu, Xiao Wan, Kaiyuan Zhu

**Affiliations:** Zhejiang Institute of Landscape Plants and Flowers, Zhejiang Academy of Agricultural Sciences, Hangzhou, 311251, Zhejiang, China

**Keywords:** *Lonicera japonica,* chloroplast genome, evolution, phylogenetic analysis

## Abstract

*Lonicera japonica* is a well-known medicinal plant, and the Damaohua cultivar is one of the oldest known honeysuckle cultivars in China. The 155,151 bp chloroplast genome of this cultivar was obtained through Illumina sequencing. The genome includes a pair of inverted repeats (IRa and IRb; 23,789 bp each), a large single-copy region (88,924 bp), and a small single-copy (SSC) region (18,649 bp). In total, 127 unique genes were identified: 80 protein-coding, 39 tRNA, and 8 rRNA genes. Only ycf3 contained two introns. Eighty-nine large repetitive sequences and 54 simple sequence repeats were detected. Fifty potential RNA editing sites were predicted. Adaptive evolution analysis revealed that infA, matK, petB, petD, rbcL, rpl16, rpl2, rps3, ycf1, and ycf2 were positively selected, possibly reflecting the specific environmental adaptations of this cultivar. Sequence alignment and analysis revealed several candidate fragments for *Lonicera* species identification, such as the intergenic regions rpoB-petN, rbcL-accD, and psaA-ycf3. The IR region boundary and phylogenetic analysis revealed that the *L. japonica* cv. Damaohua chloroplast genome was most closely related to the *L. japonica* genome, but there were five distinct differences between the two. There are four sites with high variability between *L. japonica* and *L. japonica* cv. Damaohua with nucleotide variability (Pi) greater than 0.002, including rps2-rpoC2, atpB-rbcL, ycf1, and ycf1-trnN GUU. The differences between *L. japonica* and *L. japonica* cv. Damaohua were further confirmed by the single nucleotide polymorphism sites between these two species. Therefore, this study revealed that the chloroplast genome can serve as a universal super barcode for plant identification, which can identify differences and help distinguish *Lonicera japonica* from related species. An understanding of *Lonicera japonica* cv. Damaohua chloroplast genomics and a comparative analysis of *Lonicera* species will provide a scientific basis for breeding, species identification, systematic evolution analysis, and chloroplast genetic engineering research on medicinal honeysuckle plants.

## Graphical abstract

**Figure:**
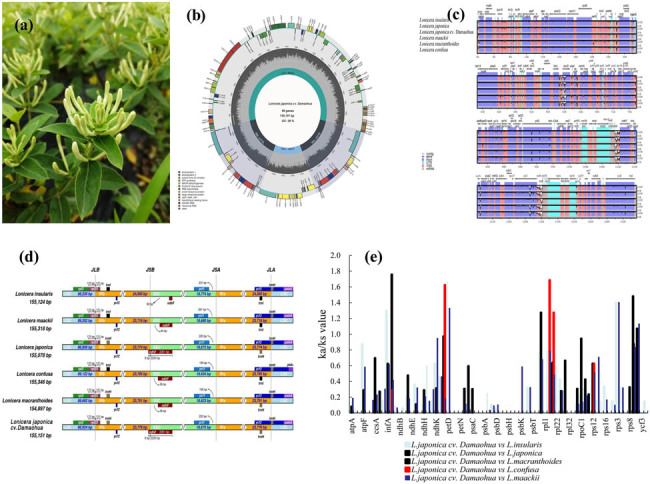

## 1 Introduction

*Lonicera japonica* Thunb. (Caprifoliaceae) is a commonly used herb that grows widely in China, Japan, Korea, and Southeast Asian countries [1]. Flower buds, known as *Lonicerae japonicae*, are the most important medicinal part of the plant [2,3], with more than 3,000 years of medical history. Lonicerae japonicae flos was first identified in one of the earliest pharmacopoeias in the world, “Shen Nong’s Material Medica,” Modern pharmacological studies have revealed that its extracts contain numerous bioactive compounds for treating various diseases, such as antibacterial, antiviral, anti-inflammatory, and antioxidative compounds [3]. In recent years, Lonicerae japonicae flos have also become the main raw material for “cooling” beverages in China.

Since the 2005 edition of the Chinese Pharmacopoeia, Lonicerae japonicae flos (called Jinyinhua, JYH in Chinese) and Lonicerae flos (called Shanyinhua, SYH in Chinese) have been documented as two herbs. *Lonicera japonica* Thunb. has been identified as the only botanical source of Lonicerae japonicae flos [4]. During the long-term cultivation process, when coupled with artificial introduction, domestication, and screening, *L. japonicae* species have undergone obvious differentiation, resulting in many intraspecific variations, such as “Damaohua,” “Xiaomaohua,” “Lanhan,” “Jiufengyihao,” “Sijihua,” and other varieties [5]. These findings indicate that the source of Lonicerae japonicae flos was more complicated. Among these varieties, *L. japonica* cv. Damaohua is one of the oldest known varieties of *L. japonicae* and is produced primarily in Shandong and Henan Provinces in China. The most prominent characteristic of this variety is its high yield; its high content of active elements (chlorogenic acid, luteoloside, flavonoids, and iridoids) is another notable feature. This variety can be considered as an excellent parental candidate [6]. Examples of its derivatives include *L. japonica* cv. Jiufengyihao and *L. japonica* cv. Beihuayihao [7,8]. There are also significant differences in the yield and quality of *L. japonica* compared with the different original species [9]. At present, there are two prominent problems associated with the application of *L. japonica*. First, the source of *L. japonica* flos on the market is complex, and it is difficult to identify the source plant based on morphological characteristics alone; thus, it is necessary to develop a more effective species identification method. Second, few studies have investigated the population inheritance of *L. japonica*, especially the intraspecific variation of the original species of traditional Chinese medicines. Therefore, research on the intraspecific variation of the original medicinal plant materials is not only helpful for understanding the process and reasons for the intraspecific differentiation of medicinal plants but also provides a theoretical basis for screening excellent germplasm resources and improving new medicinal varieties. Therefore, the accurate identification of the original botanical source is the first step in ensuring the quality of the resulting herbal medicines [10].

The identification of the original species of traditional Chinese medicines has always been a popular topic in the field of medicinal plant research. The conventional morphological characteristics and commonly used DNA barcode fragments are insufficient for identifying honeysuckle species accurately, especially for the identification of closely related species. Difficulties in species identification have led to uneven quality among medicinal honeysuckle compounds [2,11,12,13]. Genetic analysis is a reliable strategy for elucidating the evolutionary relationships among species at various taxonomic levels. Owing to the existence of functional genes that play important roles in plant cells, the chloroplast genome contains enough genetic information for comparative analysis and species diversification studies [14]. Although honeysuckle nuclear genome sequencing has been completed [15], the chloroplast genome is less common than the plant nuclear genome and has a relatively conserved structure. Chloroplast genomes have become ideal models for evolution and comparative genomic research [16]. The entire chloroplast genome can be used as a “super barcode” for species identification [17,18], which provides an essential basis for determining the evolutionary and phylogenetic relationships of medicinal plants in Caprifoliaceae (the honeysuckle family).

The chloroplast is an important organelle of green plants and plays a key role in autotrophic photosynthesis [19,20,21]. Among the three genomes present in plants, the chloroplast genome is the most conserved. The chloroplast genome is maternally inherited in most plants, and its composition and sequence are highly conserved. The chloroplast genome is a typical circular multicopy DNA molecule in cells that can be generally divided into four fragments: a large single-copy (LSC) region, a small single-copy (SSC) region, and two inverted repeat (IR) regions. The genome size usually ranges from 120 to 170 kb and includes 120–130 genes [22]. The different sizes of chloroplast genomes among different species are attributable primarily to the contraction or expansion of the IR region. Compared with the chloroplast genomes of other plants [23,24], the *L. japonica* chloroplast genome has a unique rearrangement between trnI-CAU and trnN-GUU. In the *L. japonica* chloroplast genome, rps19, rpl2, and rpl23 are moved from the IR region to the LSC region. The ycf1 pseudogene is lost from the IR region, and only one copy is present in SSC region2. These changes lead to sequence differences between species and can be used to study plant taxonomy, phylogeny, and evolutionary relationships. Genomic data, including organelle genomic data, can provide a molecular basis for research on the original species of traditional Chinese medicines [11,25].

With the increase in the number of available chloroplast genomes for Loniceraceae plants, the chloroplast genome sequences of Loniceraceae species have become easier to splice [1,2,8,26,27]. He et al. first reported the chloroplast genome sequence of *Lonicera japonica* [2]. Only the differences between plant families were compared and analyzed. Liu et al. compared the chloroplast genome sequence variation in seven *Lonicera* species [8], which was not the source of the original *L. japonica* Thunb. species specified in the Chinese Pharmacopoeia. Therefore, the phylogenetic evolution of the original species *Lonicera japonica* Thunb. is still poorly understood. There have been few reports on the chloroplast genome of *L. japonica* cv. Damaohua, which is one of the main varieties of honeysuckle in China. In general, there have been few studies on the genetics of *L. japonica* cv. Damaohua, especially the molecular genetics of this variety, which has severely restricted the protection, development and use of *L. japonica* cv. Damaohua resources.

In this study, the complete chloroplast genome of *L. japonica* cv. Damaohua was obtained using Illumina sequencing assembly. Here we not only described the whole chloroplast genome sequence of *L. japonica* cv. Damaohua and the characteristics of its long repeats and simple sequence repeats (SSRs) but also analyzed the chloroplast genome and compared it with those of other members of the original species of *L. japonica* Thunb. This study revealed that the *L. japonica* cv. Damaohua chloroplast genome was the most closely related to that of *L. japonica* from a previous report, but there were five distinct differences between the two. These results suggest that the chloroplast genome can be used to discriminate among *Lonicera* species effectively at the species level. Comprehensive chloroplast genome analysis of *L. japonica* cv. Damaohua will facilitate the identification of *Lonicera* species and provide a further basis for elucidating the evolutionary origins of this species.

## 2 Materials and methods

### 2.1 DNA sequencing, genome assembly, and annotation

**Figure 1:**
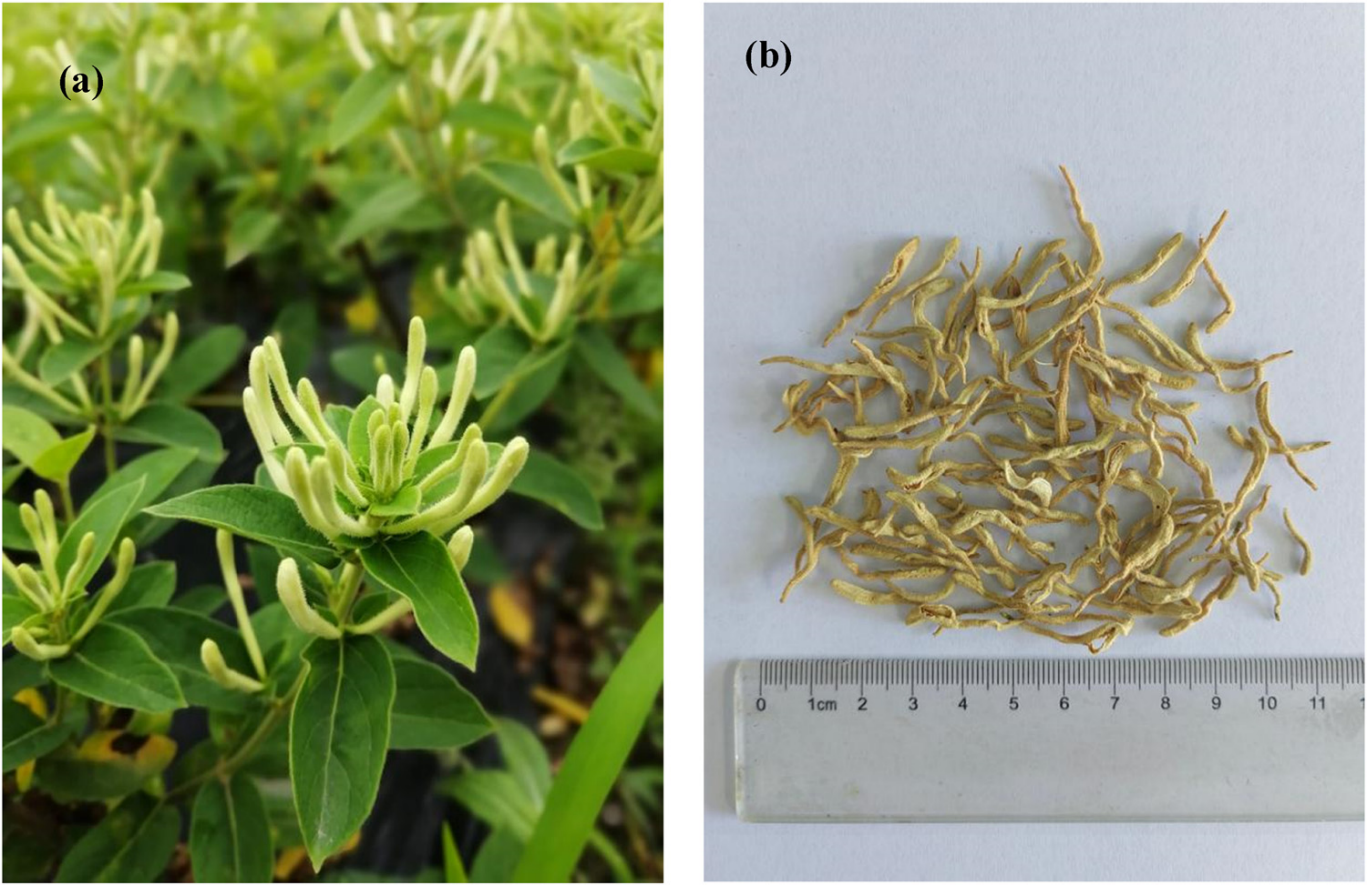
Images of *Lonicera japonica* cv. Damaohua. (a) Phenotypic characteristics of *Lonicera japonica* cv. Damaohua at the bud stage. (b) Flower buds and flowers of *Lonicera japonica* cv. Damaohua. Photographs by Jiaqiang Zhang.

Fresh leaves of *L. japonica* cv. Damaohua were collected from Hangzhou in Zhejiang Province, China (N30°06′, E120°22′) (Figure 1a and b), and total genomic DNA was extracted by cetyltrimethylammonium bromide method [28]. The DNA quality was tested (> 50 ng μL^−1^), and the DNA was then sequenced with an Illumina HiSeq 2500 platform (Illumina, San Diego, CA, USA) at Genesky Biotechnologies Inc. (Shanghai, China).

The quality of the Illumina paired-end raw reads was evaluated with FastQC software [29], and low-quality and short (*L* < 60 bp) reads were filtered. The remaining clean reads were assembled using NOVOPlasty (https://github.com/ndierckx/NOVOPlasty) [30] with the chloroplast genome sequence of *L. japonica* (NC_026839) being used as the reference sequence. The chloroplast genome was annotated using CPGAVAS2 [31]. Protein-coding genes, tRNA genes, and rRNA genes were predicted using the default parameters and were then functionally annotated in combination with the Kyoto Encyclopedia of Genes and Genomes [32,33,34], Clusters of Orthologous Groups, Nonredundant, Swiss-Prot, and Gene Ontology databases.

The chloroplast genome sequence of *L. japonica* cv. Damaohua has been submitted to GenBank (accession number: MZ779026). The associated BioProject, sequence read archive, and Bio-Sample numbers are PRJNA780957, SRR16980190, and SAMN23258802, respectively.

### 2.2 Genome structure and comparative analysis

The online tool MISA (http://pgrc.ipk gatersleben.de/misa/misa.html) [35] was used to identify SSRs in the chloroplast genome of *L. japonica* cv. Damaohua. There were ten mononucleotide repeats; five dinucleotide repeats; four trinucleotide repeats; and three tetranucleotide, pentanucleotide, and hexanucleotide repeats. Repetitive sequences, including palindromic, forward, reverse, and complement sequences, were analyzed with the REPuter program [36] with the following parameter settings: a minimal repeat size of 30 bp, a Hamming distance of 3, and a maximum number of computed repeats of 90. The relative synonymous codon usage (RSCU) value was calculated using MEGA6 [37]. RNA editing sites were predicted using the PREP-Cp web server (http://prep.unl.edu/cgi-bin/cp-input.pl) with a cutoff value of 0.8 [38]. The IR regions were examined and plotted using the online program IRscope (https://irscope.shinyapps.io/irapp/) [39]. The complete chloroplast genomes of the six *Lonicera* species were compared with the mVISTA online program using the annotation of *L. insularis* as a reference in shuffle-LAGAN mode [40]. We analyzed nonsynonymous (Ka) and synonymous (Ks) substitution rates, and the chloroplast genome sequence of *L. japonica* cv. Damaohua was compared with those of six *Lonicera* species. The coding sequences (CDSs) were subsequently translated into protein sequences. We identified 74 shared protein-coding genes that were shared between them. Ka/Ks ratio was calculated with TBtools according to the coding sequence [41]. Ka/Ks ratios > 1 indicate positive selection, Ka/Ks ratios = 1 indicate neutral selection, and Ka/Ks ratios < 1 indicate negative selection (purifying selection) [42].

We compared the chloroplast genome of *L. japonica* cv. Damaohua (MZ779026) with the chloroplast genomes of *L. macranthoides* (NC_040959), *L. confusa* (NC_045045), *L. japonica* (NC_026839), *L. maackii* (NC_039636), and *L. insularis* (MH028739).

### 2.3 Phylogenetic analysis

A total of 15 complete chloroplast genome sequences were downloaded from the NCBI database, namely, those of *L. japonica* cv. Damaohua (MZ779026), *L. macranthoides* (NC_040959), *L. confusa* (NC_045045), *L. japonica* (NC_026839), *L. maackii* (NC_039636), *L. insularis* (MH028739), *L. ferdinandi* (NC_040963), *L. hispida* (NC_040962), *L. nervosa* (NC_040961), *L. praeflorens* (NC_039635), *L. sachalinensis* (MH028742), *L. vesicaria* (NC_039638), *L. tatarica* (MW340876), *T. pinnatifidum* (NC_037952) and *C. boreale* (NC_037388). With *C. boreale* as the outgroup, the complete chloroplast genomes were aligned using MAFFT version 7 [43], and then the phylogenetic analysis was subsequently conducted using maximum likelihood (ML) and Bayesian inference (BI) methods. ML analysis was performed using the program RAxML v7.2.6 [44], BI analysis was implemented using the program MrBayes v3.1.2 [45], and visualization was performed using FigTree 1.4.4 (http://tree.bio.ed.ac.uk/software/figtree/). In addition, DnaSP v5.10 was used to evaluate the nucleotide diversity (Pi) between the *L. japonica* and *L. japonica* cv. Damaohua chloroplast genomes with a window length of 600 bp and step length of 200 bp [46].

## 3 Results

### 3.1 Chloroplast genome features of *L. japonica* cv. Damaohua

**Figure 2:**
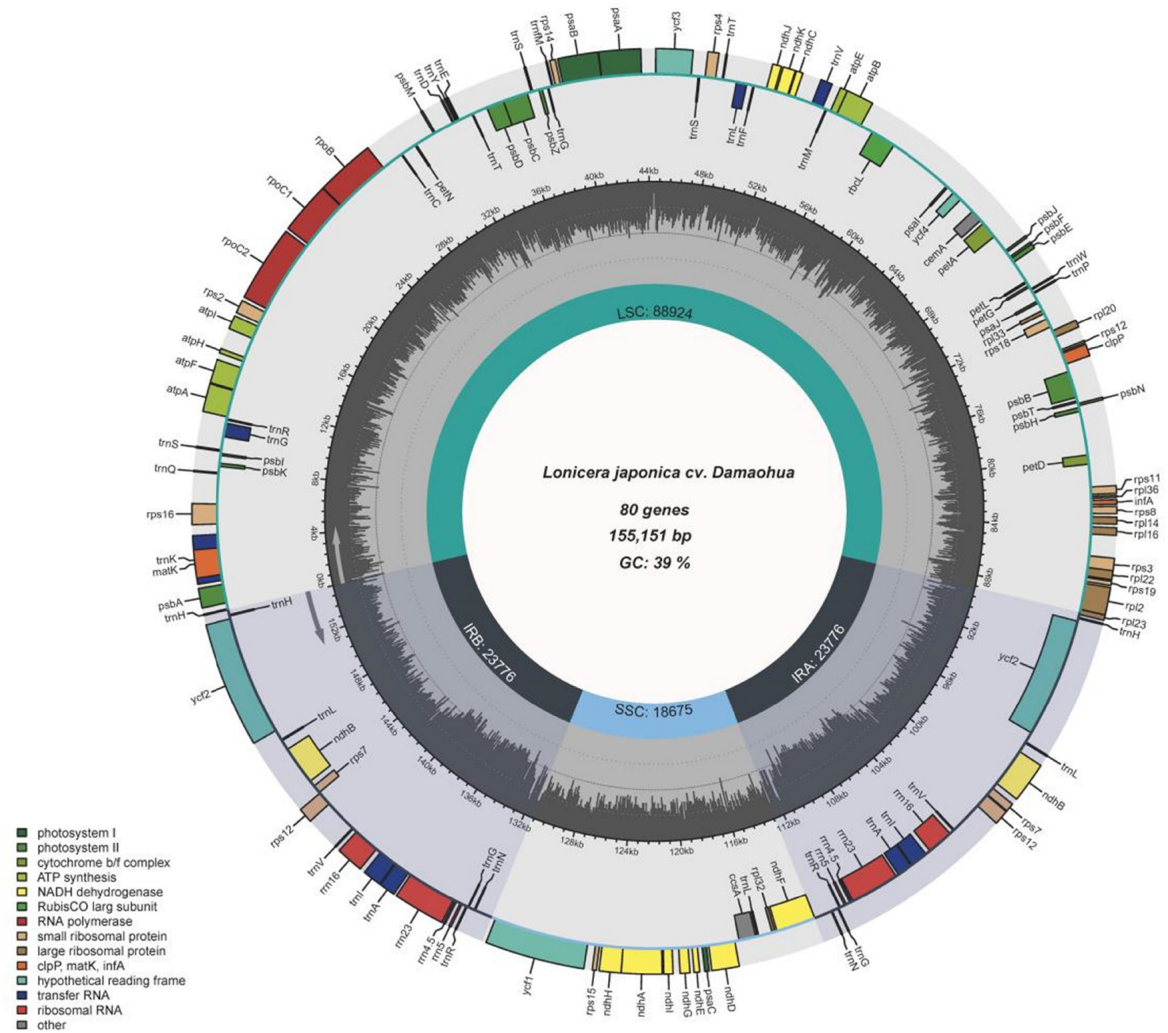
Gene map of the *L. japonica* cv. Damaohua chloroplast genome. LSC indicates large single-copy; SSC indicates small single-copy; and IR indicates inverted repeat.

The chloroplast genome sequence of *L. japonica* cv. Damaohua was found to be 155,151 bp in length, with a typical tetragonal structure consisting of an LSC region (88,924 bp), an SSC region (18,649 bp), and two IRs (IRA and IRB, 23,789 bp) (Figure 2). The chloroplast genome of *L. japonica* cv. Damaohua has 127 functional genes, consisting of 80 protein-coding genes, 8 ribosomal RNA (rRNA) genes, and 39 transfer RNA (tRNA) genes. The overall nucleotide composition of *L. japonica* cv. Damaohua was 30.2% A, 31.2% T, 19.6% C, and 19.0% G, and the total guanine (G) and cytosine (C) content was 38.6% (Table 1). A total of 16 genes were found to be repeated in the IR region, with 10 protein-coding genes (atpF, ndhA, ndhB, rps12, rps16, rps18, rpl2, rpoC1, ycf2, and ycf3) and 6 tRNA genes (trnA-UGC, trnG-GCC, trnI-GAU, trnK-UUU, trnL-UAA, and trnV-UAC). Gene structure analysis revealed that 16 genes contained introns, of which 15 (9 protein-coding genes and 6 tRNA genes) had one intron, whereas only one (ycf3) had two introns. The intron of trnK-UUU was the longest, whereas the intron of rps12 was the shortest (Table 2 and Table S1).

**Table 1:** Base composition of the *L. japonica* cv. Damaohua chloroplast genome

Region	Length	T(U) (%)	C (%)	A (%)	G (%)
Genome	155,151	31.2	19.6	30.2	19
LSC	88,924	32.1	19.0	30.8	18.1
SSC	18,649	34.3	16.9	32.3	16.5
Ira	23,789	28.7	23.1	27.9	20.4
IRb	23,789	27.9	20.4	28.7	23.1
CDS	74,214	31.3	18.1	30.0	20.6
CDS, 1 position	24,738	23.7	19.3	29.7	27.3
CDS, 2 position	24,738	32.5	20.6	28.9	18.0
CDS, 3 position	24,738	37.7	14.4	31.4	16.5

**Table 2:** Introns in the genes of the *L. japonica* cv. Damaohua chloroplast genome

GeneID	Gene	Exon Ⅰ (bp)	Intron Ⅰ (bp)	Exon Ⅱ (bp)	Intron Ⅱ (bp)	Exon Ⅲ (bp)
1	atpF	144	735	409		
2	ndhA	552	1,092	538		
3	ndhB	776	678	755		
4	rpl2	386	671	428		
5	rpoC1	431	776	1,607		
6	rps12	113	231	535	—	31
7	rps16	39	868	229		
8	rps18	160	307	30		
9	trnA-UGC	37	808	34		
10	trnG-GCC	22	716	46		
11	trnI-GAU	36	946	34		
12	trnK-UUU	36	2,534	34		
13	trnL-UAA	35	512	48		
14	trnV-UAC	39	564	35		
15	ycf2	6,118	248	483		
16	ycf3	123	731	229	759	152

We assessed the basic characteristics of the chloroplast genomes of *Lonicera*ceae and compared them with those of the *L. japonica* cv. Damaohua chloroplast genome. The *L. japonica* cv. Damaohua genome was slightly larger than the *L. japonica* (155,078 bp), *L. insularis* (155,124 bp), and *L. macranthoides* (154,897 bp) genomes but smaller than the *L. maackii* (155,318 bp) and *L. confusa* (155,346 bp) genomes (Table 3). Among the Loniceraceae chloroplast genomes, *L. macranthoides*, *L. maackii*, *L. japonica*, and *L. insularis* presented the greatest number of chloroplast genes (131), followed by *L. confusa* (129) and *L. japonica* cv. Damaohua, which presented the fewest (127). In addition, the GC contents of the chloroplast genomes from the six *Lonicera* species were similar, ranging from 38.3 to 38.6%.

**Table 3:** Comparison of the general features of the chloroplast genomes from six *Lonicera* species

Species	GenBank accession numbers	All length (bp)	LSC length (bp)	SSC length (bp)	IR length (bp)	Protein coding	tRNA LSC	rRNA IRA	Total GC (%)
*L. japonica* cv. Damaohua	MZ779026	155,151	88,924	18,675	23,776	80	39	8	38.6
*L. macranthoides*	NC_040959	154,897	88,692	18,623	23,791	84	39	8	38.5
*L. confusa*	NC_045045	155,346	89,122	18,634	23,795	84	37	8	38.6
*L. japonica*	NC_026839	155,078	88,858	18,672	23,774	84	39	8	38.6
*L. maackii*	NC_039636	155,318	89,202	18,680	23,718	86	37	8	38.5
*L. insularis*	MH028739	155,124	88,230	18,774	24,060	86	37	8	38.3

### 3.2 Characterization of SSRs and repeat sequences

A total of 54 SSRs were detected in the chloroplast genome of *L. japonica* cv. Damaohua, of which 36 were mononucleotide, 4 were dinucleotide, 2 were trinucleotide, 9 were tetranucleotide, and 3 were hexanucleotide repeats, with pentanucleotide deletions (Table 4). In addition, we compared the SSR distribution pattern and number of *L. japonica* cv. Damaohua with those of the five other chloroplast genomes in the Loniceraceae family (Tables S2 and S3). Among the six *Lonicera* species, there were more mononucleotide repeats than all the other types combined, and most of the SSRs were composed of mononucleotide and tetranucleotide repeats. The numbers and types of chloroplast SSRs vary in different species. Among the species, *L. japonica* cv. Damaohua, *L. confusa*, *L. maackii*, and *L. insularis* all lacked pentanucleotide repeats, whereas *L. macranthoides* lacked hexanucleotide repeats. *L. japonica* cv. Damaohua (54 SSRs) and *L. confusa* (54 SSRs) presented the greatest number of SSRs, whereas *L. japonica* (47 SSRs) presented the fewest SSRs. In addition, the chloroplast genomes of *L. insularis*, *L. macranthoides,* and *L. maackii* contained 52, 51, and 48 SSRs, respectively. The main type of SSR was a mononucleotide repeat, and most of the mononucleotide repeats were A/T-type SSRs.

**Table 4:** Number of different SSR types in the chloroplast genomes of six *Lonicera* species

SSR types	*L. japonica* cv. Damaohua	*L. macranthoides*	*L. confusa*	*L. japonica*	*L. maackii*	*L. insularis*
Mononucleotide	36	33	37	29	29	33
Dinucleotide	4	6	4	4	7	6
Trinucleotide	2	4	2	2	1	2
Tetranucleotide	9	7	8	9	10	9
Pentanucleotide	—	1	—	—	—	—
Hexanucleotide	3	—	3	3	1	2
Total	54	51	54	47	48	52

**Figure 3:**
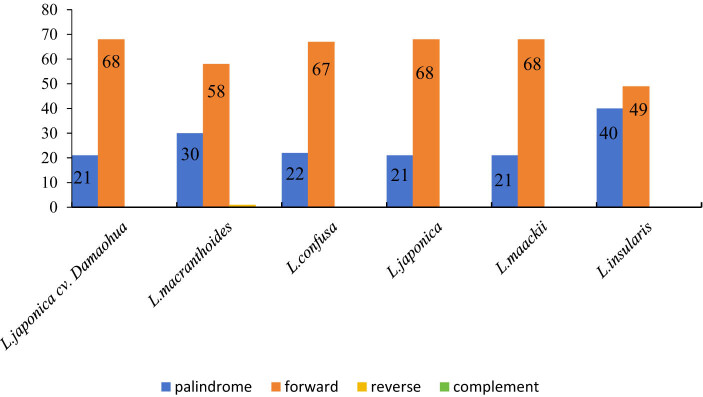
The number of different repeats in the four chloroplast genomes of the six Lonicera species. P = palindromic, F = forward, R = reverse, and C = complement.

A total of 89 large repeats were identified in the chloroplast genome of *L. japonica* cv. Damaohua using REPuter, including 68 forward repeats and 21 palindrome repeats (Figure 3). Among them, the largest repeat was a forward repeat with a size of 83 bp. The repetitive sequences of the chloroplast genomes from the six *Lonicera* species were compared and analyzed. *L. macranthoides* had palindrome, forward, and reverse repeats, whereas *L. japonica* cv. Damaohua, *L. confusa*, *L. japonica*, *L. maackii*, and *L. insularis* presented palindrome and forward repeats. *L. japonica* cv. Damaohua and *L. maackii* presented the highest number of forward repeats (68), whereas *L. insularis* presented the fewest forward repeats (49). *L. insularis* had the most palindrome repeats (40), whereas *L. japonica* cv. Damaohua and *L. maackii* had the same number of palindrome repeats (21 each) (Table S4).

### 3.3 Codon usage analysis

**Figure 4:**
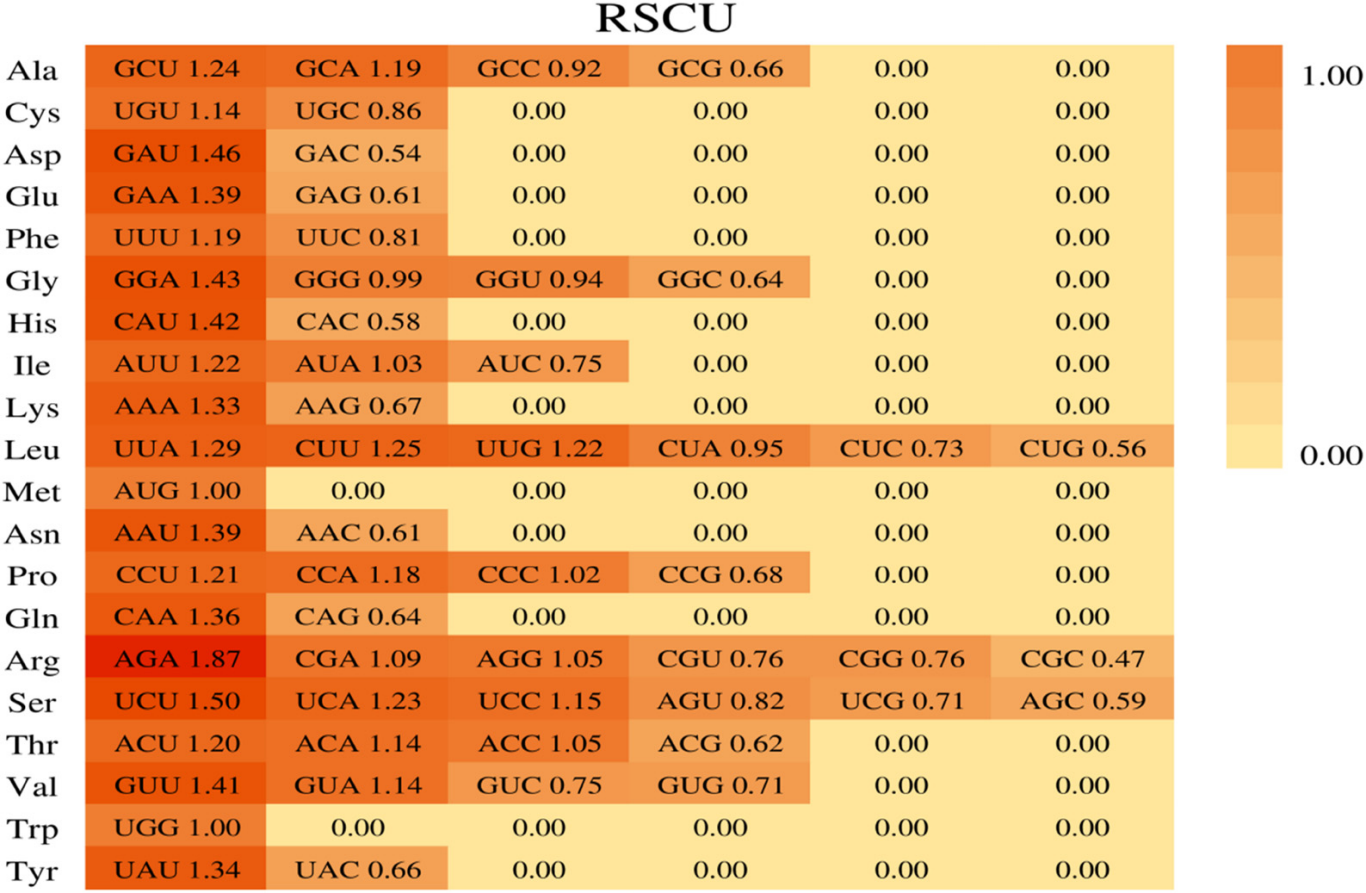
Codon contents of 20 amino acids and stop codons in all protein-coding genes of the *L. japonica* cv. Damaohua chloroplast genome. The color in the histogram corresponds to the color of the codons.

The chloroplast genome of *L. japonica* cv. Damaohua consisted of 51,717 analyzed codons. Among these codons, the most commonly used was UUU (2,132), encoding Phe, and the least commonly used was CGC (254), encoding Arg. There were 31 codons with RSCU values greater than 1 that all ended in A/U, indicating codon usage bias (Figure 4). The RSCU values of the chloroplast genomes of the six *Lonicera* species slightly differed. The six Loniceraceae species presented the highest frequency of UUU (encoding Phe) in the genome. *L. japonica* and *L. insularis* presented the lowest frequency of GCGs (encoding Ala), and *L. japonica* cv. Damaohua, *L. macranthoides*, and *L. confusa*. The least commonly used codon in the *L. maackii* genome was CGC (encoding Arg). Except Met, all the amino acids are encoded by multiple codons. Leu, Arg, and Ser have six synonymous codons; Ala, Gly, Pro, Thr, and Val have four synonymous codons; Ile, Trp, and stop codons have three synonymous codons; and Cys, Asp, Glu, Phe, His, Lys, Asn, and Gln all have two synonymous codons. In the codon usage bias analysis, *L. insularis* had 35 codons with RSCU values greater than 1, and *L. japonica* cv. Damaohua, *L. confusa*, *L. japonica*, and *L. maackii* had 33 codons with RSCU values greater than 1. *L. macranthoides* had 32 codons with RSCU values greater than 1, and most of the synonymous codons ended in A and U (Table S5).

Fifty potential RNA editing sites were found in the chloroplast genome of *L. japonica* cv. Damaohua (Table S6). The ndhD gene contained the greatest number of RNA editing sites (ten), followed by ndhB with eight editing sites; rpoB with seven editing sites; matK with four editing sites; and ndhA, petB, rpl2, and rps2 with two editing sites. The following genes had one editing site (the lowest number): atpA, atpF, atpI, ccsA, clpP, ndhF, ndhG, psaI, psbE, psbF, rpl20, rpoC2, and rps8. Among the 50 potential RNA editing sites, 10 were observed at the first position of the codon, and 40 were observed at the second position. No potential RNA editing sites were found in the third position, and the base conversion types were all C-to-T. This result is similar to those reported in other land plants. The amino acid conversion from Ser to Leu was the most frequent, whereas the conversion from Leu to Phe was the least frequent.

The RNA editing sites of the chloroplast genomes from the six *Lonicera* species were different. Among the species, *L. insularis* had the greatest number of RNA editing sites with 61, followed by *L. maackii* with 59; and *L. japonica* with the fewest 48. The accD gene is a potential RNA editing site that was not detected in *L. japonica* cv. Damaohua, *L. vesicaria,* or *L. macranthoides*, and the clpP gene was a potential RNA editing site not detected in *L. maackii* or *L. confusa*. The rpoA gene was a potential RNA editing site not detected in *L. japonica* cv. Damaohua, *L. macranthoides*, or *L. confusa*, and rpoC1 was the only potential RNA editing site detected in *L. confusa*. There were ten genes with no potential RNA editing sites detected, including atpB, petD, petG, petL, psaB, psbB, rpl23, ycf14, ycf16, and ycf3. Among the six potential RNA editing sites in Loniceraceae, most were located in the second position of the codon; no potential RNA editing sites were found in the third position. The base conversion types ranged from C to T, and Ser-to-Leu was the most frequent amino acid conversion.

### 3.4 Comparison of complete chloroplast genomes among *Lonicera* species

**Figure 5:**
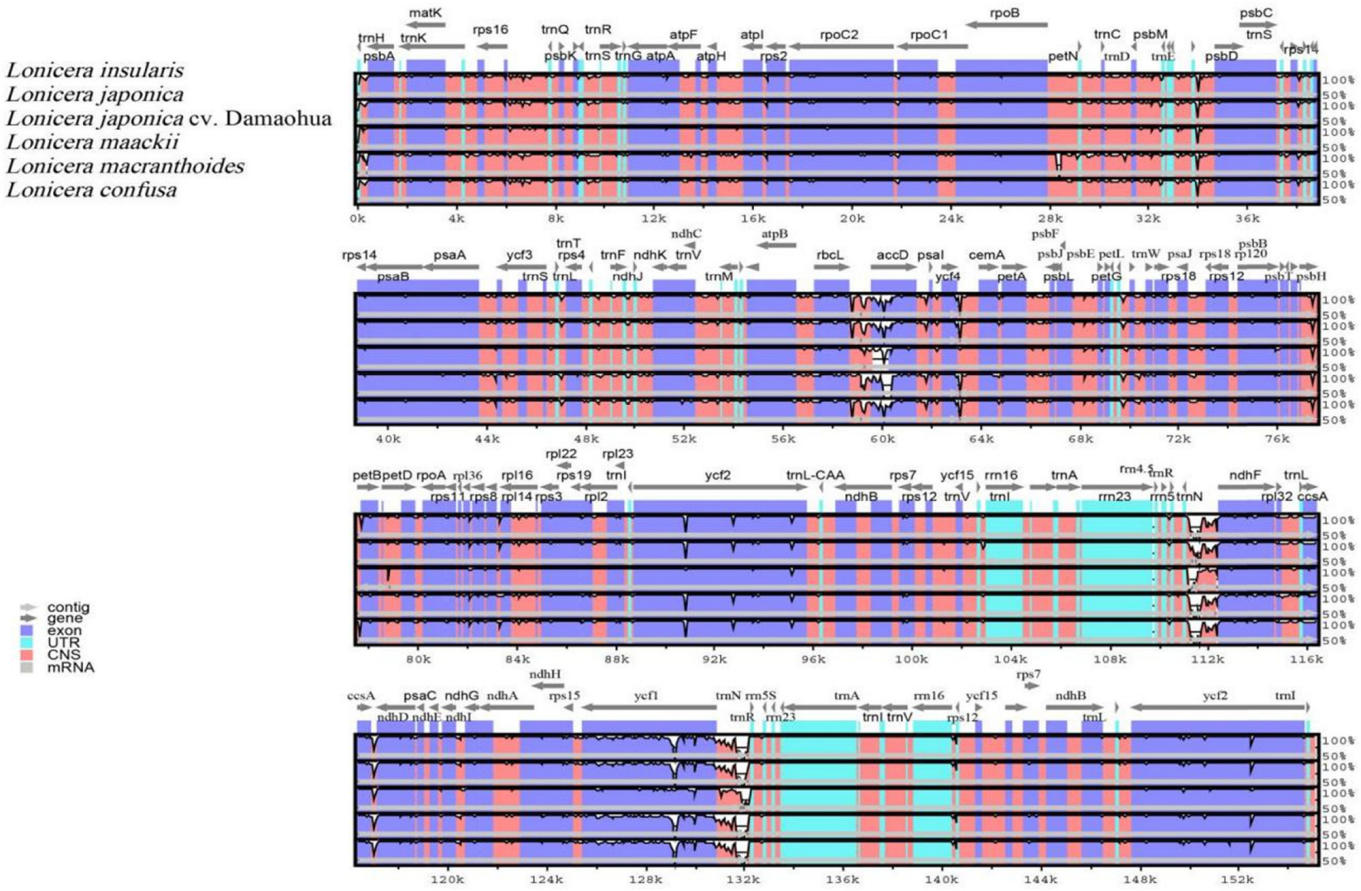
Comparison of the chloroplast genomes of six *Lonicera* species using the mVISTA program.

To characterize the genomic differences, we used the program mVISTA to align the sequences of the six Lonicera species and used the annotations of *L. insularis* as a reference. The comparison revealed that the chloroplast genomes of the six *Lonicera* species were highly similar, with very few differences (Figure 5). Compared with the LSC and SSC regions, the IR region presented fewer differences. In addition, the divergent coding regions were smaller than the noncoding regions. Among the coding genes were genes in highly conserved regions, including matK, rpoC2, rpoB, ycf1, and ycf2. In addition, several highly differentiated regions, such as the rpoB-petN, rbcL-accD, and psaA-ycf3 intergenic regions, were identified.

### 3.5 Expansion and contraction of the IR region

**Figure 6:**
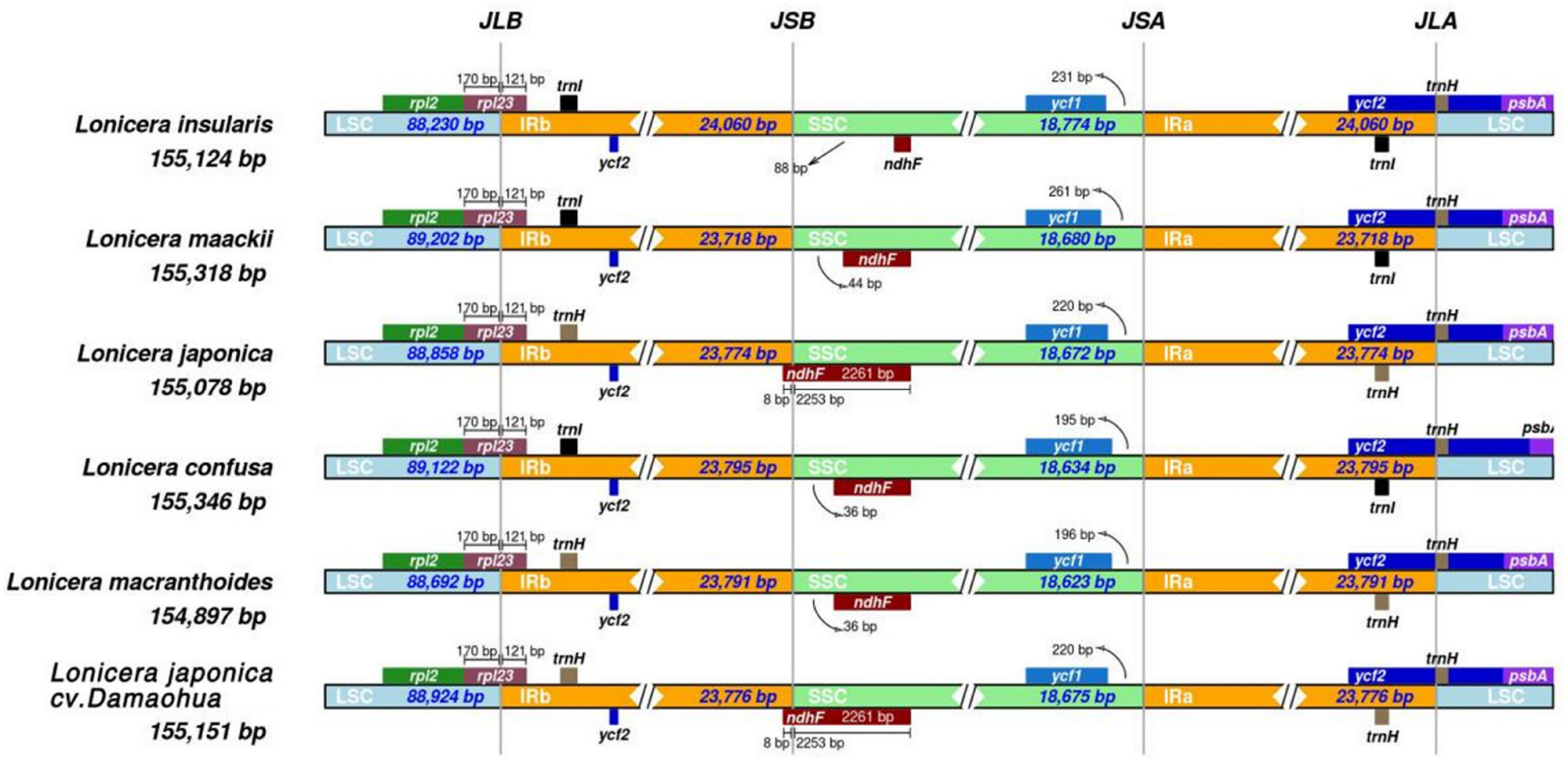
Comparison of the borders of the LSC, SSC, and IR regions among the six chloroplast genomes of Lonicera species. (JLB: junction of the LSC and IRB; JSB: junction of the SSC and IRB; JSA: junction of the SSC and IRA; and JLA: junction of the LSC and IRA).

The IR-LSC and IR-SSC boundaries of the chloroplast genome of *L. japonica* were compared with those of five reported *Lonicera* species (Figure 6). The chloroplast genomes of the six *Lonicera* species were relatively conserved, and the six boundaries of *L. japonica* cv. Damaohua and *L. japonica* were similar. The chloroplast genomes of the six *Lonicera* species were located in the coding region of rpl23 at the LSC/IRa junction. The IRb/SSC connection between IRb and the SSC region (junction of the SSC and IRB [JSB]) was located between the ycf2 and ndhF genes in four species (*L. insularis*, *L. maackii*, *L. confusa*, and *L. macranthoides*), whereas it was located in the coding area of ndhF in *L. japonica* cv. Damaohua and *L. japonica*. The ycf1 gene was located on the IRa/SSC boundary in the six *Lonicera* species, but the length of the IRa/SSC junction of ycf1 in the SSC and IRa regions was different (*L. insularis*: 231 bp; *L. maackii*: 261 bp; *L. japonica* cv. Damaohua: 220 bp; *L. confusa*: 195 bp; *L. macranthoides*: 196 bp; and *L. japonica*: 220 bp).

### 3.6 Adaptive evolution analysis

**Figure 7:**
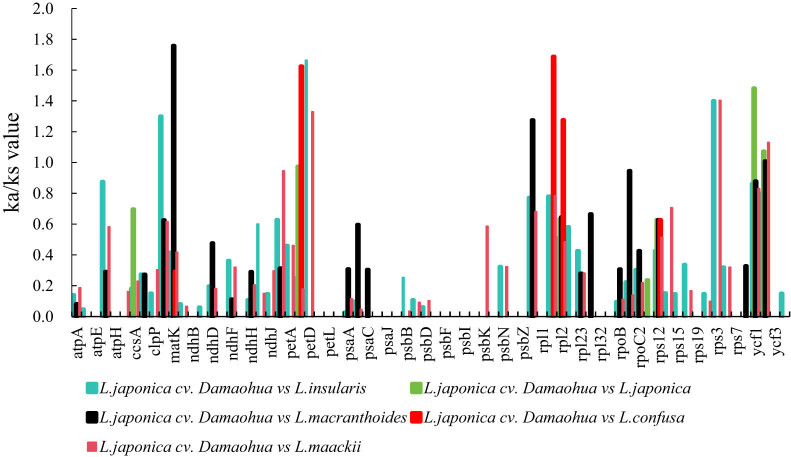
The Ka/Ks ratios of 74 protein-coding genes from the chloroplast genomes of the six Lonicera species.

TBtools was used to calculate the synonymous (Ks) and nonsynonymous (Ka) substitution rates and Ka/Ks ratios in the chloroplast genomes of the six *Lonicera* species to detect whether the 74 shared protein-coding genes were under selection pressure (Figure 7). The results revealed that the majority of genes presented Ka/Ks ratios < 1, indicating that the chloroplast genes of Loniceraceae species were subjected to purifying selection during long-term evolution (Table S7). A total of ten positively selected genes (Ka/Ks > 1) were detected in this study. Among them, the infA gene was positively selected in *L. japonica* cv. Damaohua vs *L. insularis*; the matK gene was positively selected in *L. japonica* cv. Damaohua vs *L. macranthoides*; the petB gene was positively selected in *L. japonica* cv. Damaohua vs *L. confusa*; the petD gene was positively selected in *L. japonica* cv. Damaohua vs *L. insularis*, and *L. japonica* cv. Damaohua vs *L. maackii*; the rbcL gene was positively selected in *L. japonica* cv. Damaohua vs *L. macranthoides*; the rpl16 gene was positively selected in *L. japonica* cv. Damaohua vs *L. confusa*; the rpl2 gene was positively selected in *L. japonica* cv. Damaohua vs *L. confusa*; the rps3 gene was positively selected in *L. japonica* cv. Damaohua vs *L. insularis* and *L. japonica* cv. Damaohua vs *L. maackii*; the ycf1 gene was positively selected in *L. japonica* cv. Damaohua vs *L. japonica*; and the ycf2 gene was positively selected in *L. japonica* cv. Damaohua vs *L. japonica*, *L. japonica* cv. Damaohua vs *L. macranthoides* and *L. japonica* cv. Damaohua vs *L. maackii*. These findings indicate that these genes may have undergone positive selection during evolution.

### 3.7 Phylogenetic analysis

**Figure 8:**
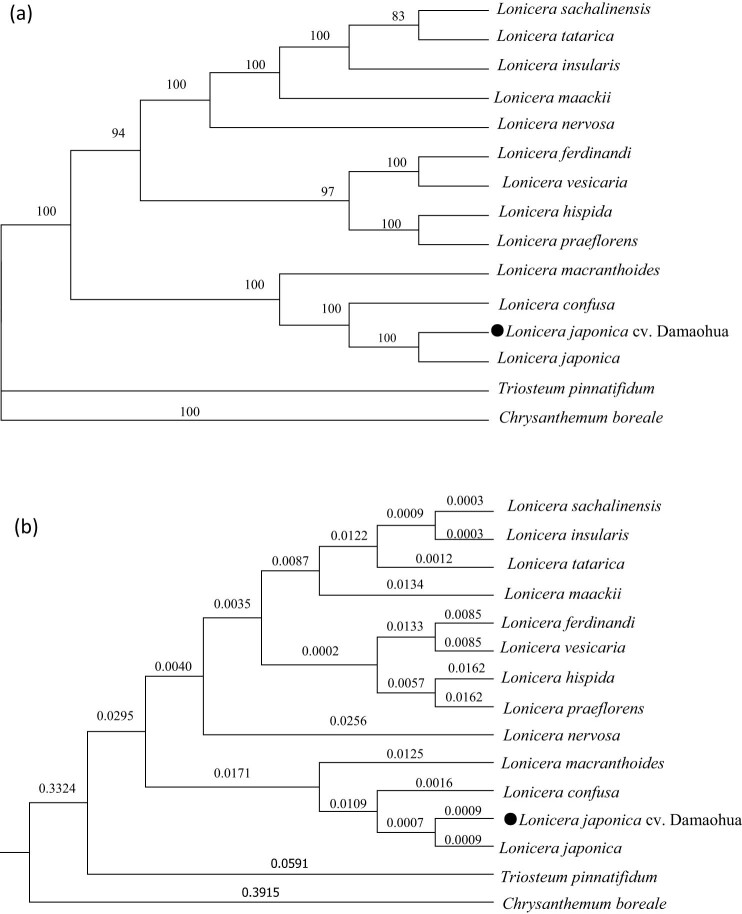
Phylogenetic analysis of Lonicera species based on the complete chloroplast genome with (a) ML and (b) BI analysis methods.

To clarify the phylogenetic positions and evolutionary relationships of Caprifoliaceae, the whole chloroplast genome sequences of 14 species of Caprifoliaceae were selected, and *Chrysanthemum boreale* was used as the outgroup to construct phylogenetic tree using the ML and BI methods (Figure 8). The ML and BI analysis results show that these two methods are consistent with one another. The chloroplast genome sequences of the 15 plant species were divided into 2 groups. The outgroup *C. boreale* was a separate group. *Triosteum pinnatifidum* and 13 *Lonicera* species were grouped. In Caprifoliaceae, Triosteum and *Lonicera* were divided into two subgroups. In *Lonicera*, the chloroplast genome sequence of *L. japonica* cv. Damaohua had the closest relationship with that of *L. japonica*, followed by *L. confusa* and *L. macranthoides*. The three phylogenetic trees of these 14 chloroplast genome sequences were consistent with traditional taxonomy, indicating that the chloroplast genome can be used to effectively analyze the phylogenetic positions and relationships of species effectively.

### 3.8 Analysis of variant loci

To further analyze the chloroplast genome differences between *L. japonica* and *L. japonica* cv. Damaohua, we used *L. japonica* as a control to compare the single nucleotide polymorphisms (SNPs) and small insertions/deletions (InDels) among the chloroplast genomes of the six *Lonicera* species (Table 5). Table 5 shows that there are 2,204 variant sites between *L. japonica* and *L. maackii*, including 1,957 SNPs and 247 InDels; there are 2,196 variant sites between *L. japonica* and *L. insularis*, including 1,931 SNPs and 265 InDels; and *L. japonica* and *L. japonica* cv. Damaohua have extremely high sequence similarity, with only 41 variant sites between them, consisting of 14 SNPs and 27 InDels.

**Table 5:** SNPs and InDels among the chloroplast genomes of the six Lonicera species

Species	*L. japonica*							
	LSC		SSC		IR		Total	
	SNP	InDel	SNP	InDel	SNP	InDel	SNP	InDel
*L. japonica* cv. Damaohua	7	20	7	5	0	2	14	27
*L. macranthoides*	419	93	105	20	106	8	630	121
*L. confusa*	49	37	9	9	7	4	65	50
*L. maackii*	1,187	170	346	38	424	39	1,957	247
*L. insularis*	1,095	181	406	34	430	50	1,931	265

### 3.9 Nucleotide diversity analysis

**Figure 9:**
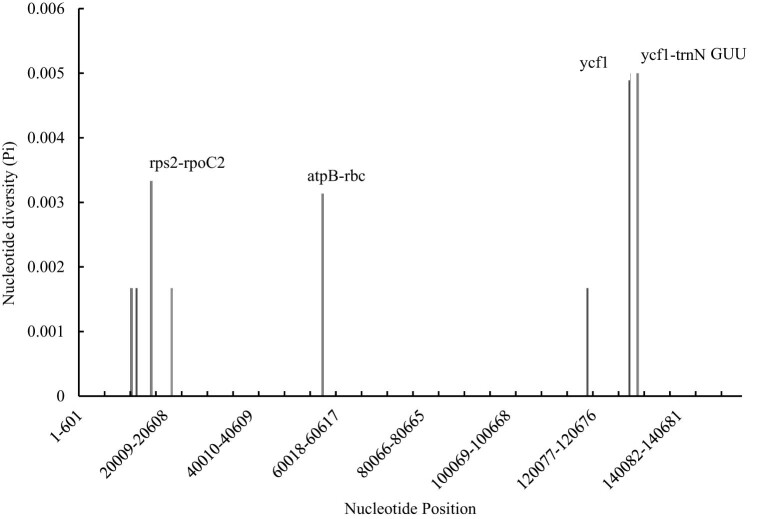
Comparison of the nucleotide variability (Pi) values between *L. japonica* and *L. japonica* cv. Damaohua.

The coding gene regions of *L. japonica* and *L. japonica* cv. Damaohua were compared, and different hotspot regions were analyzed by nucleotide variation (Pi) values (Figure 9). We obtained eight different loci (five of these regions were located in the LSC regions and three of these regions were located in the SSC regions) in the chloroplast genomes of *L. japonica* and *L. japonica* cv. Damaohua (Pi > 0.001), namely, atpA-atpF, atpF-atpH, rps2-rpoC2, rpoC2-rpoC1, atpB-rbcL, ndhD-ndhE, ycf1, and ycf1-trnN GUU. Four variable sites (Pi > 0.002) were detected between the chloroplast genomes of *L. japonica* and *L. japonica* cv. Damaohua, namely, rps2-rpoC2, atpB-rbcL, ycf1, and ycf1-trnN GUU. This finding shows that the IR region is more conserved.

## 4 Discussion

Accurate identification of the original species is the first stage in ensuring the quality of herbal medicines [11,12,13]. Owing to the variety of *Lonicera japonica* Thunb. and the complex original species, the identification of the original *Lonicera japonica* Thunb. species has not been effectively solved, which makes the protection of *Lonicera japonica* Thunb. germplasm resources and the safe use of medicinal materials inconvenient. DNA barcoding technology is stable and objective because it is not affected by environmental factors, tissue types, or developmental stages. However, DNA barcoding is often unable to distinguish among closely related species, which limits its application in species identification. The chloroplast genome contains more variation and the phylogenetic resolution is significantly greater, which is valuable for revealing phylogenetic relationships between closely related species [17,18]. In this study, the feasibility of a plant identification method based on the chloroplast genome was validated. There were some differences in the genome size and LSC, SSC, and IR regions among the six chloroplast genomes of Loniceraceae, and the number of genes and GC content were similar, reflecting the high degree of conservation of angiosperm chloroplast genomes to some extent [47,48,49]. The GC contents of the chloroplast genomes of the six *Lonicera* species were similar, ranging from 38.3 to 38.6%, and the genome size and IR, LSC, and SSC region lengths and gene contents were highly similar across the chloroplast genomes, indicating that the gene structure of *Lonicera* was highly conserved.

Introns play important roles in RNA stability, gene expression regulation, and alternative splicing, which have been reported in many other species [50]. He et al. reported that the ycf3 gene and the rps18 gene contained two introns in *L. japonica* [2]. In this study, the ycf3 gene included two introns and rps18 had only one intron in the chloroplast genome of *L. japonica* cv. Damaohua. The ycf3 gene is necessary for the accumulation of the photosystem I (PSI) complex and acts as a chaperone that interacts with the PSI subunit at the posttranslational level [51]. This finding revealed a large amount of intron gain and/or intron loss during the evolution of Loniceraceae [52].

Comparative analysis of chloroplast genomes is an important method for deep investigation of complex evolutionary [53,54]. The mVISTA program was used to analyze the differences in the chloroplast genomes of the six *Lonicera* species. The comparison revealed that the chloroplast genomes of *Lonicera* species were highly conserved, but there was a certain degree of variation. The coding region was more conserved than the noncoding region, the IR region was more conserved than the LSC and SSC regions, and there were more variations in the latter two regions than in other regions. Similar findings have been reported in the chloroplast genomes of certain genera in previous studies [11,55,56]. The mutation levels of the matK, rpoC2, rpoB, ycf1, and ycf2 genes were slightly lower, while the mutation levels of the rpoB-petN, rbcL-accD, and psaA-ycf3 gene regions were quite different. These regions are sources of potential barcodes for the identification of *Lonicera* species. Owing to its standardization, minimization, and scalability, DNA barcoding has become an extremely widely used technique in molecular marker-based species identification. At present, DNA barcoding technology has also made some progress in the identification of honeysuckle medicinal plants. These six divergence hotspot regions (trnH-GUG-psbA, rps2-rpoC2, rbcL-psaI, trnN-GUU-ndhF, rps15-ycf1, and infA) should be applied to the development of molecular markers in *Lonicera* species. Studies have shown that the species identification efficiency of DNA barcoding was closely related to the species selected and the quantity. In this study, rpoB-petN, rbcL-accD, and psaA-ycf3 gene regions were highly obvious. These markers were potential barcode candidate gene. And these DNA barcoding candidate genes have also been identified in other species. The rpoB-petN can be used as DNA barcode for the identification of *Sanicula L.* [57]. The rbcL-accD was used for the identification of the Fritillaria germplasm resources [58]. Several studies have verified the use of hotspot regions of the chloroplast genome for identifying species. Sequencing results proved that psaA-ycf3 can be used as a specific DNA barcode to accurately identify *Zizania* spp. [59], *Fosbergia* [60], *Streptocarpus ionanthus* [61], and Fertile Lycoris [62]. The divergent regions of rbcL-accD and psaA-ycf3 were highly obvious, which could be used as DNA barcodes for the taxonomic evidence of Stellaria dichotoma [63]. In this study, these potential barcodes require further experimental validation.

Ka/Ks analysis is an effective method for assessing whether the functions of protein-coding genes have evolved. Synonymous nucleotide substitutions occur more frequently than nonsynonymous substitutions for most genes, so the Ka/Ks value is usually less than 1 [64,65,66]. A total of ten positively selected genes (including infA, matK, petB, petD, rbcL, rpl16, rpl2, rps3, ycf1, and ycf2) were detected in this study. This finding indicates that most of the *Lonicera* genes are under negative selection and that only ten genes have undergone positive selection. These ten genes included two subunits of cytochrome b/f complex genes (petB and petD), two large subunit of ribosome genes (rpl16 and rpl2), subunit of rubisco gene (rbcL), translational initiation factor gene (infA), maturase gene (matK), and ycf1 and ycf2. The petB encodes a protein containing S1 domain in photosynthetic electron transfer B, which is associated with in the stability and translation of chloroplast mRNAs [67]. The petD constitutes a subunit of cytochrome b/f complex, which is critical to electron flow. It is useful to noting that in the response of plants to drought stress, half of the chloroplasts revealed a decrease in petD [68]. The rpl16 and rpl2 genes encode ribosomal proteins, which are indispensable for chloroplast translation devices [69]. The rbcL gene encodes the Rubisco subunit. Rubisco catalyzes the assimilation of carbon dioxide in the atmosphere during photosynthesis. Positive selection of rbcL is quite common in terrestrial plants [70]. The positive selection of rbcL in *Lonicera* species is linked to adaptation to low carbon dioxide concentrations in high-altitude environments. The infA are genes involved in photosynthesis, responsible for encoding and translating starting factors. The infA gene was absent in the chloroplast genome of some plant, which may be involved in the transfer of functional genes to the nuclear genome, leading to their replacement by nuclear-encoded proteins [69,71,72]. The saturation enzyme K protein, encoded by the matK gene, is the only recognized group II intrinsic maturation enzyme in chloroplast genome. The mature enzyme matK is required to split its own and other group II introns and plays a role in photosynthesis and plant development. The products encoded by the genes ycf1 and ycf2, which are essential in the chloroplast genome of higher plants, are indispensable for cell survival [70]. Among these genes, ycf2 presented Ka/Ks values > 1 in three of the species, indicating that this gene has recently undergone evolution. Ycf2 is a gene with a coding region in the chloroplast genome. The function of its encoded product is still unclear. The ycf2 gene is positively selected in many plants. It has been speculated that this gene plays an important role in the adaptation of terrestrial plants to environmental changes. Therefore, this positively selected gene in *L. japonica* cv. Damaohua may help this cultivar to adapt its specific environment.

The phylogenetic tree constructed based on the complete chloroplast genome has greater support and discrimination ability. The use of the complete chloroplast genome as a super-barcode to accurately identify closely related species in an accurate manner is therefore proposed [17,18]. In this study, we found that the *L. japonica* cv. Damaohua chloroplast genome was most closely related to that of the previously reported *L. japonica*, but there were some clear differences between them. The first difference was the chloroplast genome size and the number of encoded proteins. The *L. japonica* chloroplast genome is 155,078 bp in length and contains 84 protein-coding genes. The chloroplast genome of *L. japonica* cv. Damaohua is 155,151 bp in length and includes 80 protein-coding genes. The second difference was the number of genes that had two introns. *L. japonica* cv. Damaohua contains only one gene (ycf3), whereas *L. japonica* contains two genes (rps18 and ycf3). The third difference was the difference in the number of SSRs. *L. japonica* cv. Damaohua found 54 SSRs. In *L. japonica*, 47 SSRs were found. The fourth difference was that the ycf1 and ycf2 genes were positively selected in *L. japonica* cv. Damaohua vs *L. japonica*. The fifth difference was that there were 14 SNPs and 27 InDels that differed between *L. japonica* and *L. japonica* cv. Damaohua. The chloroplast genome is characterized by maternal inheritance and is highly conserved in composition and sequence. These results suggest that *L. japonica* cv. Damaohua and *L. japonica* are different, and that they may be related species. The chloroplast genome can be used to effectively discriminate among intraspecific variations effectively in *L. japonica* from related species. The super-barcode is a useful complement to current molecular identification methods. These findings provide an important basis for determining the evolutionary and phylogenetic relationships of medicinal plants in the *Lonicera* species.

The chloroplast genome contains a large amount of genetic information and has become a powerful tool for species identification and phylogenetic research on medicinal plants [48,49,52,53,54]. Compared with the commonly used DNA barcode fragments, the chloroplast genome can provide more informative loci [61,62,63]. In this study, the complete chloroplast genome of *L. japonica* cv. Damaohua was obtained using Illumina sequencing assembly and those of five other species of honeysuckle SNP sites were screened and identified SNP sites, and further established molecular identification methods for related species were established. Among them, four different mutation hotspots (rps2-rpoC2, atpB-rbcL, ycf1, and ycf1-trnN GUU) with high nucleotide variability (Pi > 0.002) could serve as a new and unique DNA barcode for honeysuckle and its related species. Because of the increasing demands for *Lonicera japonica* in the herbal market, quality control has become an important issue for this variety [1,11,18]. Thus, an authentic DNA marker to identify these species could be utilized to prevent adulteration or abuse other *Lonicera* species. Therefore, we hope to develop more DNA markers and establish a practical *Lonicera* species authentication system. Based on the results of this study, the next step is to develop a DNA barcoding method for the identification of *Lonicera* and its related species.

The availability of the complete *L. japonica* cv. Damaohua chloroplast genome provides sequence information that can be used to confirm the phylogenetic location of *L. japonica* cv. Damaohua and understand phylogenetic relationships between Loniceraceae species. However, since we used only a few species in the Loniceraceae family, we should further study other chloroplast genomes and nuclear genome sequences of *Lonicera* species to provide more sufficient evidence to explain the evolutionary process of Caprifoliaceae accurately.

## 5 Conclusion

In this study, the chloroplast genome of *L. japonica* cv. Damaohua was sequenced and assembled, providing a valuable genome resource for *Lonicera*. A comparative analysis of *L. japonica* cv. Damaohua and its relatives revealed that the chloroplast genomes of the six *Lonicera* species were highly similar, with very few differences. Compared with the LSC and SSC regions, the IR region presented fewer differences. In addition, the divergent coding regions were smaller than the noncoding regions. Phylogenetic analysis of the chloroplast genome revealed that *L. japonica* cv. Damaohua and *L. japonica* had a close intra-genus relationship, but there were five distinct differences between the two. Four sites with high variability with a nucleotide variability (Pi) greater than 0.002 between *L. japonica* and *L. japonica* cv. Damaohua, including rps2-rpoC2, atpB-rbcL, ycf1, and ycf1-trnN GUU were located. These different SNP sites between these two species further confirmed the differences between the species. These results suggest that the chloroplast genome can be used to discriminate intraspecific variation in *L. japonica* from related species effectively. This finding also verifies the feasibility of using the chloroplast genome as a super-barcode to identify *L. japonica* and related species. These findings provide a basis for future research on the genetic resource development, species identification and conservation biology of *Lonicera*.

## Supplementary Material

Supplementary Table
